# Azole Antifungal Sensitivity of Sterol 14α-Demethylase (CYP51) and CYP5218 from *Malassezia globosa*

**DOI:** 10.1038/srep27690

**Published:** 2016-06-13

**Authors:** Andrew G. S. Warrilow, Claire L. Price, Josie E. Parker, Nicola J. Rolley, Christopher J. Smyrniotis, David D. Hughes, Vera Thoss, W. David Nes, Diane E. Kelly, Theodore R. Holman, Steven L. Kelly

**Affiliations:** 1 Centre for Cytochrome P450 Biodiversity, Institute of Life Science, Swansea University Medical School, Swansea, Wales SA2 8PP, United Kingdom; 2 Chemistry and Biochemistry Department, University of California, Santa Cruz, CA 95064 USA; 3 Plant Chemistry Group, School of Chemistry, Bangor University, Bangor, Gwynedd, Wales, LL57 2UW, United Kingdom; 4 Center for Chemical Biology, Department of Chemistry and Biochemistry, Texas Tech University, Lubbock, Texas 79409-1061.

## Abstract

*Malassezia globosa* cytochromes P450 CYP51 and CYP5218 are sterol 14α-demethylase (the target of azole antifungals) and a putative fatty acid metabolism protein (and a potential azole drug target), respectively. Lanosterol, eburicol and obtusifoliol bound to CYP51 with *K*_d_ values of 32, 23 and 28 μM, respectively, catalyzing sterol 14α-demethylation with respective turnover numbers of 1.7 min^−1^, 5.6 min^−1^ and 3.4 min^−1^. CYP5218 bound a range of fatty acids with linoleic acid binding strongest (*K*_d_ 36 μM), although no metabolism could be detected in reconstitution assays or role in growth on lipids. Clotrimazole, fluconazole, itraconazole, ketoconazole, voriconazole and ketaminazole bound tightly to CYP51 (*K*_d_ ≤ 2 to 11 nM). In contrast, fluconazole did not bind to CYP5218, voriconazole and ketaminazole bound weakly (*K*_d_ ~107 and ~12 μM), whereas ketoconazole, clotrimazole and itraconazole bound strongest to CYP5218 (*K*_d_ ~1.6, 0.5 and 0.4 μM) indicating CYP5218 to be only a secondary target of azole antifungals. IC_50_ determinations confirmed *M. globosa* CYP51 was strongly inhibited by azole antifungals (0.15 to 0.35 μM). MIC_100_ studies showed itraconazole should be considered as an alternative to ketoconazole given the potency and safety profiles and the CYP51 assay system can be used in structure-activity studies in drug development.

*Malassezia* species are known to play a role in several human skin diseases including dandruff, seborrheic dermatitis, pityriasis versicolor and malassezia folliculitis and may also exacerbate atopic dermatitis and psoriasis^1^,^2^,^3^,^4^,^5^ even though they are members of the normal skin microbial flora, being present on the skin of 75 to 98% of healthy individuals^6^. There are presently fourteen recognized species of *Malassezia*, eight of which are associated with humans^7^. *Malassezia* are unique amongst fungi in requiring exogenous lipids for growth. *Malassezia* species have evolved through an expansion of lipid hydroxylases (14 lipases in *M. globosa*) and a reduction in carbohydrate metabolism genes which may have contributed to the evolution of the genus from plant to animal inhabitants^8^. The predominant species found in dandruff and seborrheic dermatitis sufferers are elevated levels of *M. globosa* and *M. restricta*^9^,^10^ preferring sebum-rich areas of skin, degrading the sebum by secreting lipases^11^. Dandruff may affect 50% of individuals and the more severe seborrheic dermatitis affects 1 to 3% of the population^3^,^4^.

The most commonly used antifungal agents to treat topical *M. globosa* infections are ketoconazole^12^,^13^ targeting sterol 14α-demethylase (CYP51), hydroxypiridones^3^,^14^,^15^,^16^,^17^, especially ciclopiroxolamine^17^ which targets a variety of metabolic processes including depletion of trivalent metal cations^18^ and zinc pyrithione^19^,^20^ which causes elevated copper levels in yeast cells^21^. Alternate drug targets in *M. globosa* being evaluated include β-carbonic anhydrase inhibition by sulfonamide, sulfamates and sulfamide drugs^22^.

Azole antifungal agents selectively target fungal CYP51 enzymes over the human homolog^23^ through the direct coordination of the triazole ring N-4 nitrogen (fluconazole, itraconazole and voriconazole) or the imadazole ring N-3 nitrogen (clotrimazole and ketoconazole) with the heme iron as the sixth axial ligand^24^. Azole antifungals can also coordinate with the heme iron of other cytochrome P450 enzymes, raising the possibility of alternative and secondary drug targets. CYP52 proteins are known to be involved in growth on alkanes and fatty acids in yeasts and a homolog has been described in *Malassezia*^25^ that might represent a drug target to inhibit growth on these carbon sources.

Many systemic and superficial fungal infections are also associated with tissue inflammation involving human 5-lipoxygenase (5-LOX), such as dandruff 26. Therefore a therapeutic agent that possessed both antifungal and anti-inflammatory activity would be advantageous. The drug candidate ketaminazole^27^, possessing antifungal activity through an imidazole moiety and anti-inflammatory action through a phenylenediamine moiety, was shown to be a potent inhibitor of *Candida albicans* CYP51 and human 5-LOX. In this study, the expression, purification and characterization of two *M. globosa* cytochrome P450 monooxygenase proteins (CYP51 and CYP5218) are described and the effectiveness of azole antifungal agents against *M. globosa* CYP51 and CYP512 including inhibition of *M. globosa* growth are determined.

## Results

### Purification of CYP51 and CYP5218 proteins

**Figure 1:**
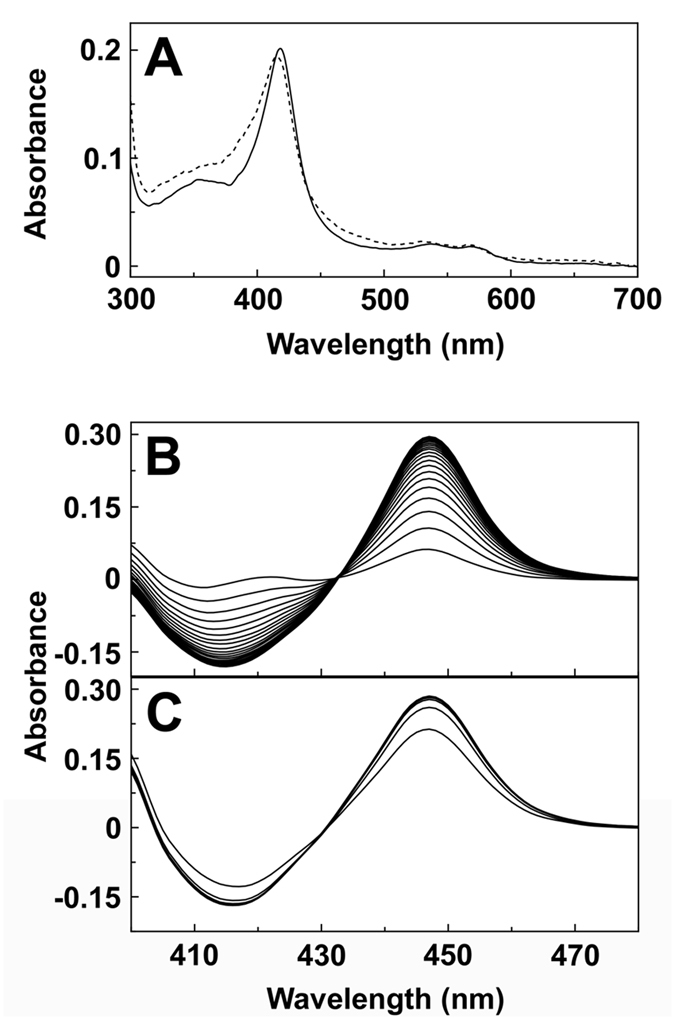
Spectral characteristics of *M. globosa* CYP51 and CYP5218. Absolute spectra (**A**) were determined using 3 μM purified *M. globosa* CYP51 (solid line) and CYP5218 (dashed line) in the oxidised resting state (light path 4.5 mm). Reduced carbon monoxide difference spectra were determined using 3 μM purified *M. globosa* CYP51 (**B**) and CYP5218 (**C**) with sequential measurements made every 45 seconds (light path 10 mm).

Cholate extraction yielded ~320 (±100) and ~120 (±30) nmoles per liter culture of CYP51 and CYP5218. Purification by Ni^2+^-NTA agarose chromatography resulted in 61% and 66% recoveries of native *M. globosa* CYP51 and CYP5218 proteins. SDS polyacrylamide gel electrophoresis confirmed the purity to be greater than 90% when assessed by staining intensity. The apparent molecular weights of *M. globosa* CYP51 and CYP5218 proteins were 53000 (±3000) and 58000 (±4000), which were slightly lower than the predicted values of 59786 and 69833, including N-terminal modifications and 4-Histidine C-terminal extensions. The absolute spectra of the resting oxidized form of *M. globosa* CYP51 and CYP5218 (Fig. 1A) were typical for low-spin ferric cytochrome P450 enzymes predominantly in the low-spin state^28^,^29^ with α, β, Soret (γ) and δ spectral bands at 571, 538, 418 and 360 nm for CYP51 and 565, 529, 415, 363 nm for CYP5218. Reduced carbon monoxide difference spectra for CYP51 and CYP5218 (Fig. 1B,C) gave the red-shifted heme Soret peak at 447 nm characteristic of P450 enzymes, indicating both proteins were expressed in the native form.

### Substrate binding properties

**Figure 2:**
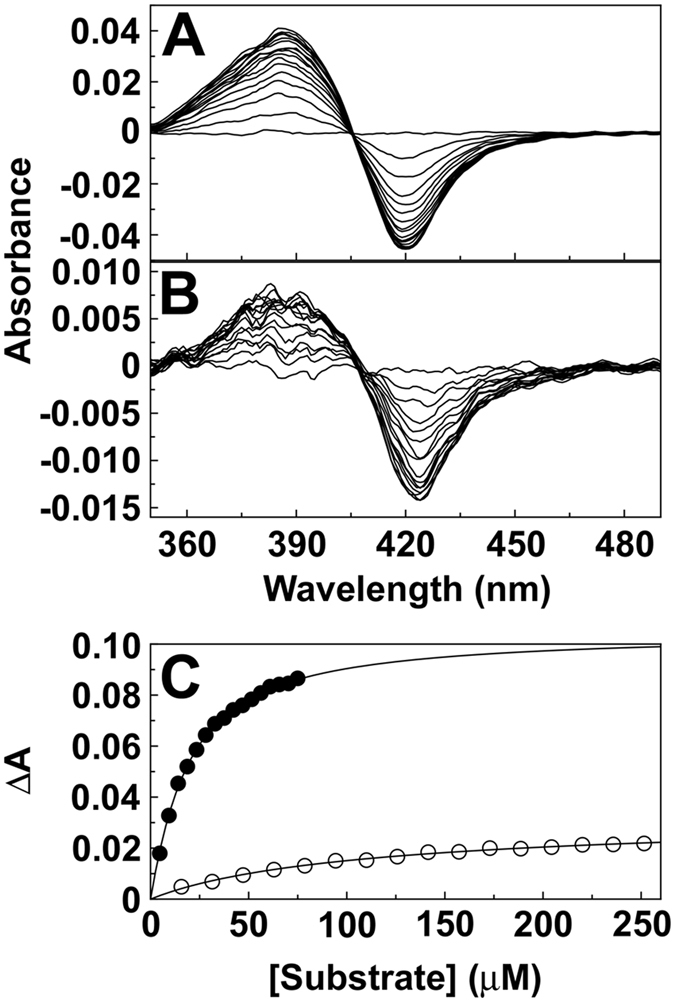
Type I substrate binding spectra. Absorbance difference spectra obtained by the progressive titration of 5 μM CYP51 with eburicol (**A**) and 4 μM CYP5218 with palmitoleic acid (**B**) were measured. Saturation curves (**C**) for eburicol (filled circles) and palmitoleic acid (hollow circles) were constructed from the absorbance difference spectra. Ligand binding data were fitted using the Michaelis-Menten equation with each experiment performed in triplicate although only one replicate is shown.

Progressive titration of *M. globosa* CYP51 with eburicol gave a type I difference spectrum with a peak at 388 nm and a trough at 421 nm (Fig. 2A). Similar spectra were obtained using lanosterol and obtusifoliol. Type I binding spectra occur when the substrate or another molecule displaces the water molecule coordinated as the sixth ligand to the low-spin hexa-coordinated heme prosthetic group causing the heme to adopt the high-spin penta-coordinated conformation^29^. Binding affinities for the three sterols were similar with *K*_d_ values of 32, 23 and 28 μM for lanosterol, eburicol and obtusifoliol, respectively.

Progressive titration of CYP5218 with palmitoleic acid gave a type I difference spectrum (Fig. 2B). The binding spectra obtained with the other fatty acids are shown in supplementary Figure S1. The binding affinities for most fatty acids with CYP5218 were similar (*K*_d_ = 90 to 250 μM) except for linoleic acid (*K*_d_ = 36 μM), which bound 2.5- to 7-fold more tightly to CYP5218 (Table 1) and with similar affinity to sterols binding to *M. globosa* CYP51. No binding spectra were obtained for CYP5218 with capric acid (C10:0), arachidic acid (C20:0), n-hexadecane and 1-hexadecene, indicating saturated fatty acids shorter than C12 and larger than C18 in addition to alkanes and alkenes did not bind to CYP5218 by perturbing the heme environment. Lanosterol and eburicol both failed to give reproducible binding spectra with CYP5218 (data not shown), excluding sterols as potential substrates.

### CYP reconstitution assays

GC/MS analysis was used to identify CYP51 and CYP5218 assay metabolites. CYP51 assays using 0.5 μM *M. globossa* CYP51 and 50 μM obtusifoliol gave a turnover number of 3.4 min^−1^ compared with 1.7 min^−1^ for lanosterol and 5.6 min^−1^ for eburicol indicating *M. globosa* CYP51 had been isolated in a fully functional form exhibiting a preference for eburicol and obtusifoliol as substrate over lanosterol. Product identities were confirmed by the mass fragmentation patterns (Figures S2–S4) to be 14-demethylated sterols. Enzyme velocity curves for 0.13 μM *M. globossa* CYP51 (Figure S5) indicated substrate inhibition at higher sterol concentrations, especially for obtusifoliol and eburicol. Calculated *K*_cat_ values of 22.47 ± 2.19, 65.54 ± 16.05 and 53.69 ± 14.21 min^−1^ for lanosterol, eburicol and obtusifoliol confirm an apparent catalytic preference for eburicol and obtusifoliol over lanosterol, however, *K*_m_ values were similar for all three sterols at 55.14 ± 6.51, 55.91 ± 19.79 and 42.09 ± 10.45 μM, respectively.

Reconstitution assays with CYP5218 resulted in no observed product formation using lauric acid, myristic acid, palmitic acid, palmitoleic acid, oleic acid and linoleic acid as substrates. This was despite increasing the duration of the assay from 1 hour to overnight (~16 h) and using three different cytochrome P450 reductase redox partners. Therefore fatty acids of chain length C12 to C18 were not CYP5218 substrates.

### Azole binding properties and IC_50_ determinations

**Figure 3:**
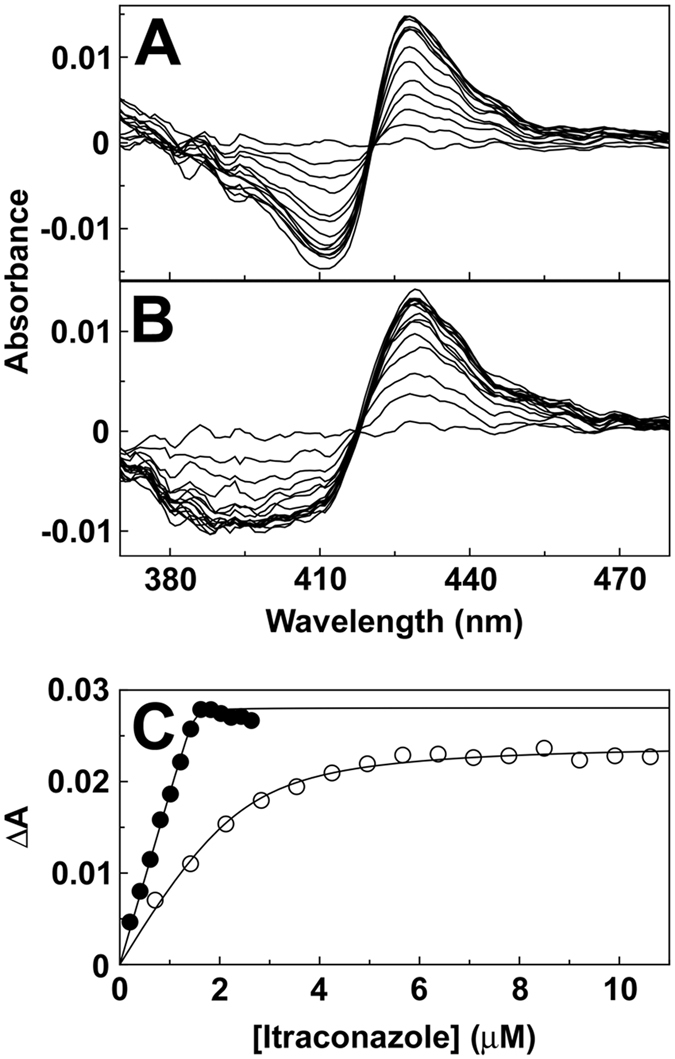
Type II itraconazole binding spectra. Itraconazole was progressively titrated against 2 μM CYP51 (**A**) and 2 μM CYP5218 (**B**) with the difference spectra determined after each addition of triazole. Itraconazole saturation curves (**C**) were constructed from the type II absorbance difference spectra for CYP51 (filled circles) and CYP5218 (hollow circles). The data were fitted using a rearrangement of the Morrison equation^47^. Each experiment was performed in triplicate although only one replicate is shown.

Titration of *M. globosa* CYP51 and CYP5218 with itraconazole produced type II binding spectra (Fig. 3A,B) with a peak at ~429 nm and a trough at ~412 nm. The binding spectra obtained with clotrimazole, fluconazole, ketoconazole, voriconazole and the drug candidate ketaminazole are shown in supplementary Figure S6. Type II binding spectra are caused by the triazole ring N-4 nitrogen (fluconazole, itraconazole and voriconazole) or the imidazole ring N-3 nitrogen (clotrimazole, ketoconazole and ketaminazole) coordinating as the sixth ligand with the heme iron^24^ to form the low-spin CYP51-azole complex resulting in a ‘red-shift’ of the heme Soret peak. All six azole antifungals bound to *M. globosa* CYP51 (Figure S6), however, fluconazole did not bind to *M. globosa* CYP5218 whereas clotrimazole, voriconazole, itraconazole, ketoconazole and ketaminazole all gave type II binding spectra with CYP5218.

Binding saturation curves (Fig. 3C and supplementary Figure S7) confirmed that azole binding to CYP51 was very tight with *K*_d_ values of 4 ± 2, 11 ± 4, 2 ± 1, 2 ± 1, 8 ± 5 and 3 ± 1 nM for clotrimazole, fluconazole, itraconazole, ketoconazole, voriconazole and ketaminazole, respectively. Azole binding to CYP5218 was weaker with itraconazole and clotrimazole binding the tightest (*K*_d_ 407 ± 136 and 486 ± 28 nM). Binding affinity to CYP5218 progressively fell from ketoconazole, ketaminazole and voriconazole (*K*_d_ values ~1.6 ± 0.2, ~11.7 ± 0.5 and ~107 ± 2.2 μM) suggesting that these azoles would be ineffective at inhibiting CYP5218 activity.

**Figure 4:**
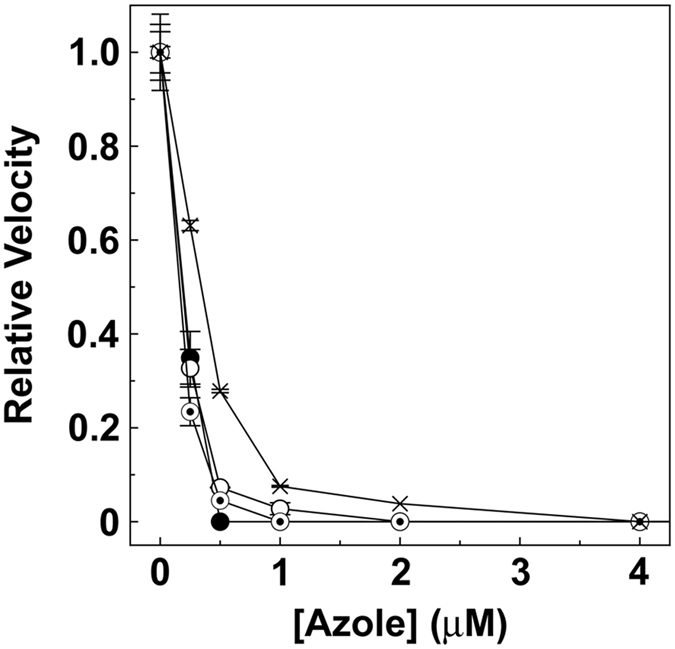
IC_50_ determinations for azole antifungal agents. CYP51 reconstitution assays were performed using a CYP51:AfCPR1 ratio of 1:2 for 0.5 μM *M. globosa* CYP51 with 50 μM lanosterol as substrate at varying fluconazole (filled circles) itraconazole (hollow circles), ketoconazole (bullets) and ketaminazole (crosses) concentrations from 0 to 4 μM. Mean relative velocity values are shown along with standard deviation bars. Relative velocities of 1.00 were equivalent to velocities of 1.79 ± 0.26 min^−1^.

IC_50_ determinations (Fig. 4) confirmed that *M. globosa* CYP51 bound fluconazole, itraconazole, ketoconazole and ketaminazole tightly with IC_50_ values approximately half the CYP51 concentration present at 0.206 ± 0.008, 0.188 ± 0.008, 0.176 ± 0.016 and 0.321 ± 0.042 μM, respectively. Similarly tight binding azole IC_50_ values were observed for *C. albicans* CYP51^23^. The ketaminazole IC_50_ value was less than two-fold higher than the current therapeutic azole drugs and was 45-fold lower than the IC_50_ value determined for ketaminazole with human CYP51^27^, suggesting ketaminazole could be an effective antifungal agent against *M. globosa* with minimal inhibition of human CYP51.

### *M. globosa* MIC determinations

Both itraconazole and ketoconazole strongly inhibited *M. globosa* growth with MIC_100_ values of 0.0625 and 0.25 μg ml^−1^. Voriconazole was moderately effective with a MIC_100_ value of 1 μg ml^−1^ whereas clotrimazole, ketaminazole and fluconazole were poor inhibitors of *M. globosa* growth (MIC_100_ values of 8, 8, and 64 μg ml^−1^). The high MIC_100_ value for fluconazole (64 μg ml^−1^) compared to the low IC_50_ for fluconazole with *M. globosa* CYP51 (0.2 μM) suggests rapid efflux of this azole from cells. Ketaminazole was 32-fold less effective than ketoconazole at arresting *M. globosa* growth and 128-fold less effective than itraconazole, however, partial inhibition of growth was observed with 4 μg ml^−1^ ketaminazole. Therefore ketaminazole appears substantially less effective at inhibiting *M. globosa* growth than itraconazole and ketoconazole given the two-fold difference in the CYP51 IC_50_ values. The treatment of *M. globosa* with 0.125 μg ml^−1^ ketoconazole and 4 μg ml^−1^ ketaminazole resulted in the accumulation of eburicol (Table 2). Accumulation of CYP51 substrates is indicative of direct *in vivo* CYP51 inhibition along with a reduction in the abundance of other post-CYP51 sterol metabolites.

## Discussion

Phylogenetic analysis of MGL_2415 strongly suggested this gene encoded a CYP51 enzyme. All 23 conserved CYP51 residues^30^ were present in the amino acid sequence and the top 100 BLASTP matches were predominantly CYP51 enzymes. In contrast, a similar analysis for MGL_3996 (CYP5218) failed to identify a putative catalytic function for this cytochrome P450 enzyme. Previously Lee *et al*.^25^ had reported that MGL_3996 was probably a CYP52 enzyme. BLAST2 analyses of CYP5218 against other yeast CYP52s showed less than 40% sequence identity. Such low sequence identities between CYP52 proteins and MGL_3996 suggest CYP5218 is not a CYP52 enzyme.

The spectral properties of purified *M. globosa* CYP51 and CYP5218 suggest both proteins were expressed in their native low-spin states. This was further supported by CYP51 binding 14-methylated sterols and CYP5218 binding fatty acids (Fig. 2). The *M. globosa* CYP51 *K*_d_ values for lanosterol and eburicol were similar to those obtained with *Candida albicans* CYP51^31^, *Mycosphaerella graminicola* CYP51^32^, and *Aspergillus fumigatus* CYP51B^33^, but were 20-fold larger than *K*_d_ values obtained with *Trypanosoma cruzei* CYP51^34^. Eburicol, lanosterol and obtusifoliol were demethylated by *M. globosa* CYP51 confirming the enzyme was isolated in native form. The catalytic rates observed with *M. globosa* CYP51 were similar to those of other CYP51 enzymes^35^. Previously *M. globosa* CYP51 was expressed and purified by Kim *et al*.^36^, but they did not demonstrate catalytic turnover.

Type I fatty acid binding spectra with *M. globosa* CYP5218 suggested that this enzyme metabolized fatty acids (hydroxylation or desaturation) with linoleic acid (C18:2) being favored because of the significantly lower *K*_d_ value (36 μM) and therefore might be implicated in the use of fatty acids as carbon source for growth.

The fatty acid binding *K*_d_ values observed for CYP5218 were similar to those determined for *Candida maltosa* CYP52A4 with lauric and myristic acids (~110 and ~120 μM)^37^. Lee *et al*.^25^ had previously expressed CYP5218 in *Pichia pastoris* at low levels, but did not purify or characterize the enzyme in terms of substrate binding properties or catalytic activity. Despite CYP5218 binding fatty acids, no *in vitro* metabolism of lauric acid, myristic acid, palmitic acid, palmitoleic acid, oleic acid or linoleic acid could be detected using the CYP5218 reconstitution assay with AfCPR1, CaCPR or HsCPR as redox partners. This suggests that fatty acids are not the *in vivo* substrates of CYP5218 in *M. globosa*. This phenomenon has previous been observed with cytochrome P450 BM3 mutants^38^. The fatty acids entered the enlarged substrate binding channel/pocket displacing the axial hexa-coordinated heme water molecule causing a low- to high-spin state change but did not undergo metabolism as the fatty acid molecule remained too distant from the heme iron for catalysis to occur.

Azole ligand binding studies indicated that *M. globosa* CYP51 bound azole antifungal agents 120- to 13300-fold more tightly than CYP5218 based on *K*_d_ values. Therefore azole antifungals would need to be redesigned if CYP5218 was to be the primary drug target. At present only itraconazole and clotrimazole appear to have reasonably high affinities for CYP5218, albeit with 200- and 120-fold higher *K*_d_ values than for CYP51. The drug candidate ketaminazole bound tightly to *M. globosa* CYP51 (*K*_d_ 3 nM) suggesting ketaminazole would be effective at inhibiting CYP51 activity and *M. globosa* growth. In comparison, *C. albicans* CYP51 had *K*_d_ values for pharmaceutical azoles of 10 to 56 nM^23^ and a *K*_d_ value of 43 nM with ketaminazole^27^. In contrast, *H. sapiens* CYP51^23^,^39^ had higher *K*_d_ values of 42 nM to 70 μM for pharmaceutical azoles and a *K*_d_ value of 731 nM for ketaminazole^27^. Therefore ketaminazole exhibited a 240-fold selectivity for *M. globosa* CYP51 over the human homolog based on *K*_d_ values compared to a 17-fold selectivity for *C. albicans* CYP51^27^.

IC_50_ determinations (Fig. 4) confirmed that *M. globosa* CYP51 was strongly inhibited by azole antifungal agents (IC_50_ values of 0.15 to 0.35 μM), suggesting efficacy against *M. globosa* cultures. However, the effectiveness of azole antifungals at inhibiting *M. globosa* cell growth was variable with itraconazole and ketoconazole being effective (MIC_100_ values 0.0625 and 0.25 μg ml^−1^) and voriconazole being moderately effective (MIC_100_ 1 μg ml^−1^), whereas clotrimazole, ketaminazole and fluconazole were poor inhibitors of *M. globosa* growth (MIC_100_ values 8, 8 and 64 μg ml^−1^). The mode of action for the azole antifungals against *M. globosa* was confirmed by sterol analysis of 0.125 μg ml^−1^ ketoconazole- and 4 μg ml^−1^ ketaminazole-treated cell pellets which showed raised eburicol levels compared to untreated cells (Table 2). Accumulation of 14-methylated sterols, such as eburicol, is characteristic of *in vivo* CYP51 inhibition by azole antifungal agents. The increased susceptibility of ketaminazole to both hydrolysis and oxidative stress in comparison to ketoconazole may partially explain the higher MIC_100_ values observed with ketaminazole when using broth-cultured *M. globosa*. The drug transporter inhibitor FK-506 was found to be cytotoxic towards *M. globosa* at concentrations above 0.0625 μg ml^−1^ preventing the assessment of the contribution of drug transporters towards azole resistance observed with ketaminazole relative to ketoconazole. Further investigations into the reduced potency of ketaminazole, especially in comparison to itraconazole and ketconazole, are required to establish whether altering the structure of the compound can overcome these apparent shortcomings. The MIC_100_ values determined in this study were towards the upper end of the MIC values previously reported of 0.016 to 0.25 μg ml^−1^ for itraconazole, 0.03 to 0.06 μg ml^−1^ for voriconazole, 0.008 to 0.25 μg ml^−1^ for ketoconazole, 0.06 μg ml^−1^ for posaconazole and 4 μg ml^−1^ for fluconazole^40^,^41^,^42^,^43^.

In summary, *M. globosa* CYP5218 was observed to bind fatty acids but was not associated with metabolism and its function and potential as a drug target remains unclear. In contrast the known drug target CYP51 readily catalyzed the 14α-demethylation of lanosterol, eburicol and obtusifoliol and bound all six pharmaceutical azoles tightly (*K*_d_ 2 to 11 nM) reflected in the low IC_50_ values for azole antifungals. The performance of the drug candidate ketaminazole against *M. globosa* CYP51 was encouraging with a *K*_d_ value similar to the other pharmaceutical azoles and an IC_50_ value only twice that of ketoconazole. However, MIC_100_ studies showed ketaminazole to be ~32-fold less effective at inhibiting *M. globosa* growth than ketoconazole, suggesting that the chemical structure of ketaminazole needs further optimization for increased cellular uptake and antifungal potency *in vivo*. A CYP51 assay was demonstrated that can be used for drug discovery and a case demonstrated for using itraconazole given the superior safety profile over ketoconazole.

## Materials and Methods

### Heterologous expression and purification of recombinant proteins

The *M. globosa* CYP51 gene (Mglob51 - KEGG *M. globosa* genome database http://www.genome.jp/dbget-bin/www_bget?mgl:MGL_2415) and CYP5218 gene (Mglob5218 - http://www.genome.jp/dbget-bin/www_bget?mgl:MGL_3996) were synthesized by Eurofins MWG Operon (Ebersberg, Germany) and cloned into the pCWori^+^ expression vector. The first eight amino acids of each protein were changed to ‘MALLLAVF’^44^ and a four-histidine tag added to the C’ terminus. The pCWori^*+*^:Mglob51 and pCWori^+^:Mglob5218 constructs were transformed into DH5α *E. coli* cells. *M. globosa* CYP51 was expressed as previously described^31^ whilst for CYP5218 the expression temperature was lowered to 20 °C for 18 h at 180 rpm. Recombinant proteins were isolated according to the method of Arase *et al*.^45^ and solubilized CYP51 and CYP5218 proteins were purified using Ni^2+^-NTA agarose^28^. Protein purity was assessed by SDS polyacrylamide gel electrophoresis.

### Cytochrome P450 spectroscopy

Reduced carbon monoxide difference spectroscopy^46^,^47^ and absolute spectra between 700 and 300 nm were used to determine cytochrome P450 concentrations. Substrate and azole binding studies were performed^31^,^48^ using 2.5 mM stock sterol solutions in 40% (wt/vol) (2-hydroxypropyl)-β-cyclodextrin (HPCD), 4 mg ml^−1^ solutions of fatty acids in dimethylformamide (DMF), and stock 0.05, 0.1 and 0.2 mg ml^−1^ solutions of azole antifungal agents in dimethylsulfoxide (DMSO). Sterols were titrated against 5 μM CYP51, fatty acids were titrated against 4 μM CYP5218 and azole antifungal agents were titrated against 2 μM CYP51 and CYP5218. The absorbance difference spectrum between 500 and 350 nm was determined after each incremental addition of ligand with saturation curves constructed from ΔA_peak-trough_ against ligand concentration. The dissociation constants (*K*_d_) for sterols and fatty acids were determined by non-linear regression (Levenberg-Marquardt algorithm) using the Michaelis-Menten equation. The dissociation constants of the enzyme-azole complex were determined by non-linear regression (Levenberg-Marquardt algorithm) using a rearrangement of the Morrison equation for tight ligand binding^49^,^50^. ProFit 6.1.12 (QuantumSoft, Zurich, Switzerland) was used for curve-fitting data. All ligand-binding studies were performed at 22 ± 2 °C and in triplicate. All spectral determinations were made using a Hitachi U-3310 UV/VIS spectrophotometer (San Jose, California).

### CYP reconstitution assays

CYP51 reconstitution assays^34^,^51^ contained 0.5 μM *M. globosa* CYP51, 1 μM *Aspergillus fumigatus* cytochrome P450 reductase isoenzyme 1 (AfCPR1 - UniProtKB accession number Q4WM67^52^), 50 μM 14α-methylated sterol (lanosterol, eburicol or obtusifoliol), 50 μM dilaurylphosphatidylcholine, 4% (wt/vol) HPCD, 0.4 mg ml^−1^ isocitrate dehydrogenase, 25 mM trisodium isocitrate, 50 mM NaCl, 5 mM MgCl_2_ and 40 mM MOPS (pH ~7.2). Assay mixtures were incubated at 37 °C for 10 minutes prior to initiation with 4 mM β-NADPHNa_4_ followed by shaking at 37 °C for 20 minutes. Sterol metabolites were recovered by extraction with ethyl acetate followed by derivatization with 0.1 ml *N*,*O*-bis(trimethylsilyl)trifluoroacetamide (BSTFA) : trimethylchlorosilane (TMCS) (99:1) and 0.3 ml anhydrous pyridine (2 h at 80 °C) prior to analysis by gas chromatography mass spectrometry (GC/MS)^53^. IC_50_ determinations were performed using 50 μM lanosterol as substrate to which azole antifungal agents were added in 2.5 μl dimethylsulfoxide prior to incubation at 37 °C and addition of β-NADPHNa_4_. Determination of *K*_cat_ and *K*_m_ values utilized CYP51 reconstitution assays that contained 0.13 μM *M. globosa* CYP51 and 0.5 μM AfCPR1 at sterol substrate concentrations of 6.25, 12.5, 25, 50, 75 and 100 μM with incubation at 37 °C for 5 min.

The CYP5218 fatty acid reconstitution assay system used was based on that previously described by Guengerich^54^ and contained 200 nM of CYP5218, 500 nM of cytochrome P450 reductase (*A. fumigatus* CPR1^52^) or *H. sapiens* CPR^55^ or *C. albicans* CPR^56^, 50 μM DLPC, 100 μM fatty acid (lauric acid, myristic acid, palmitic acid, palmitoleic acid, oleic acid and linoleic acid), 4 mM glucose-6-phosphate, 3 U/ml yeast glucose-6-phosphate dehydrogenase in a final volume of 500 μl with 0.1 M potassium phosphate (pH 7.4). Assay mixtures were incubated at 37 °C for 5 min prior to initiation with 4 mM β-NADPHNa_4_ and then incubated for a further 60 min or overnight (~16 h) at 37 °C. Fatty acid metabolites were recovered by extraction with dichloromethane and dried in a vacuum centrifuge. Samples were derivatized with 200 μl anhydrous pyridine and 250 μl BSTFA:TMCS (99:1) (30 min at 80 °C) and extracted with 500 μl of hexane, prior to analysis by GC/MS (Thermo GC Trace 1300 and ISQ MS). GC analysis was performed with a 5% phenyl methylpolysiloxane column (DB5) (Agilent Technologies). The oven programme was as follows: initial: 70 °C, with 3 min hold; ramp: 10 °C min^−1^ to 180 °C (held for 2 min), 10 °C min^−1^ to 250 °C (held for 2 min) and 10 °C min^−1^ to 280 °C (held for 2 min). Data were analyzed using Thermo Xcalibur 2.2 software.

### *M. globosa* MIC_100_ determination

*M. globosa* ATCC MYA-4612 was grown for 7 days at 30 °C in modified Dixon medium (3.6% malt extract, 2% desiccated ox-bile, 1% Tween 40, 0.6% peptone, 0.2% glycerol, 0.2% oleic acid, pH adjusted to 6 with HCl) until a culture density of 1.5 × 10^5^ cells ml^−1^ was obtained. Cells from 3 ml of stock culture were harvested, suspended in 1 ml of saline and used to inoculate 30 ml of fresh modified Dixon medium for use as *M. globosa* inoculums in MIC determinations. Stock azole concentrations of 1600, 800, 400, 200, 100, 50, 25, 12.5, 6.25, 3.125 and 0 μg ml^−1^ were prepared in DMSO. These were diluted ten-fold with fresh modified Dixon media and 20 μl of the diluted azole solutions were then mixed with 180 μl of *M. globosa* inoculum in microtiter plate wells to yield final azole concentrations of 16, 8, 4, 2, 1, 0.5, 0.25, 0.125, 0.0625, 0.03125 and 0 μg ml^−1^. The microtiter plates were incubated at 30 °C for 7 days and then read. Each azole MIC_100_ determination was performed in triplicate.

### Sterol analysis

*M. globosa* was grown in the absence and presence of azole antifungal for 7 days at 30 °C and non-saponifiable lipids extracted as previously reported^57^ and were derivatized with 0.1 ml BSTFA:TMCS (99:1) and 0.3 ml anhydrous pyridine (2 h at 80 °C) prior to analysis by GC/MS^53^. Individual sterols were identified by reference to relative retention times, mass ions and fragmentation patterns. Sterol composition was calculated using peak areas.

### Chemicals

All fatty acids were obtained from Sigma-Aldrich (Poole, UK), Fluconazole, itraconazole and ketoconazole were obtained from Sigma-Aldrich (Poole, UK) whilst ketaminazole was supplied by professor T.R. Holman (University of California, Santa Cruz, CA). Lanosterol, eburicol and obtusifoliol were supplied by professor W. D. Nes (Texas Tech University, Lubbock, TX).

## Additional Information

**How to cite this article**: Warrilow, A. G. S. *et al*. Azole Antifungal Sensitivity of Sterol 14α-Demethylase (CYP51) and CYP5218 from *Malassezia globosa*. *Sci. Rep.* **6**, 27690; doi: 10.1038/srep27690 (2016).

## Supplementary Material

Supplementary Information

## Figures and Tables

**Table 1:** Fatty acid binding affinities for *M. globosa* CYP5218.

Fatty acid	CYP5218 *K*_d_ (μM)	
	Michaelis-Menten	Eadie-Hofstee
Capric acid	no binding	no binding
Lauric acid	136 ± 4	136 ± 3
Myristic acid	195 ± 41	245 ± 29
Palmitic acid	1020 ± 755^a^	253 ± 77^a^
Stearic acid	no fit^a^	no fit^a^
Arachidic acid	no binding	no binding
Lauroleic acid	poor fit	148 ± 20
Myristoleic acid	158 ± 20	151 ± 8
Palmitoleic acid	113 ± 7	114 ± 5
Oleic acid	93 ± 11	90 ± 16
Linoelic acid	36 ± 4	36 ± 6
Arachidonic acid	264 ± 34	256 ± 36

*M. globosa* CYP5218 (4 μM) was progressively titrated with fatty acids. Saturation curves were constructed from the absorbance difference ΔA_peak-trough_ of the type I binding spectra obtained. The Michaelis-Menten equation was used to fit the ligand binding data in addition to Eadie-Hofstee analysis. Mean *K*_d_ values from three replicates are shown along with standard deviations.

^a^precipitation of fatty acid was apparent.

**Table 2:** Sterol composition of azole-treated *M. globosa*.

Sterols	Azole-treated *M. globosa* sterol fraction (%)		
	Untreated	0.125 μg ml^−1^ Ketoconazole	4 μg ml^−1^ Ketaminazole
Cholesterol^a^	1.31	1.92	1.21
Ergosterol	50.33	51.50	54.64
Ergosta-8,22-dienol	9.06	1.89	5.35
Fecosterol	0.31	0.98	0.54
Ergosta-8-enol	3.23	2.23	0
Ergosta-5,7-dienol	17.40	9.39	13.85
Episterol	0.91	0	0.63
Ergosta-7-enol	8.65	1.58	5.00
Obtusifoliol	2.22	0.18	3.52
4,4-dimethylzymosterol	2.37	0	0.62
Eburicol	4.20	30.33	14.63

^a^cholesterol assimilated from growth media containing ox-bile.
