# Supplementary material for: Azole Antifungal Sensitivity of Sterol 14α-Demethylase (CYP51) and CYP5218 from Malassezia globosa
Source: Sci Rep. 2016 Jun 13;6:27690. doi: 10.1038/srep27690 (PMC4904373; doi:10.1038/srep27690)
Supplement: Supplementary Information [file srep27690-s1.pdf]

Supplementary Information:

Azole Antifungal Sensitivity of Sterol 14α-Demethylase (CYP51) and CYP5218 from *Malassezia globosa*

Andrew G.S. Warrilow[1], Claire L. Price[1], Josie E. Parker[1], Nicola J. Rolley[1], Christopher J. Smyrniotis[2], David D. Hughes[3], Vera Thoss[3], W. David Nes[4], Diane E. Kelly[1], Theodore R. Holman[2] and Steven L. Kelly[1*]

[1]Centre for Cytochrome P450 Biodiversity, Institute of Life Science, Swansea University Medical School, Swansea, Wales SA2 8PP, United Kingdom. [2]Chemistry and Biochemistry Department, University of California, Santa Cruz, CA 95064 USA. [3]Plant Chemistry Group, School of Chemistry, Bangor University, Bangor, Gwynedd, Wales, LL57 2UW,United Kingdom. [4]Center for Chemical Biology, Department of Chemistry and Biochemistry, Texas Tech University, Lubbock, Texas 79409-1061.

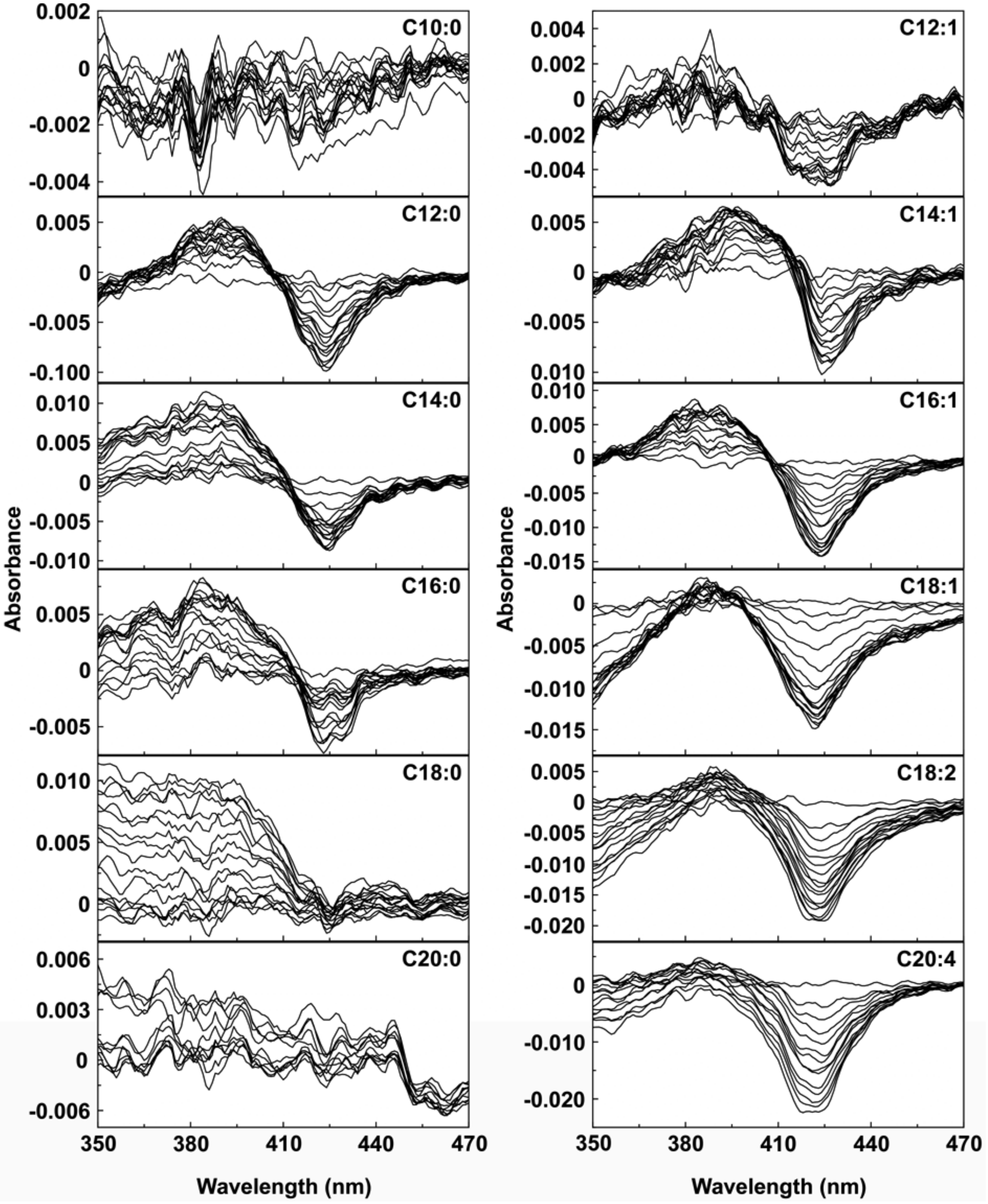

Figure S1. Fatty acid binding properties of *M. globosa* CYP5218. Absorbance difference spectra were measured during the progressive titration of 4 µM CYP5218 with the fatty acids C10:0 (capric acid), C12:0 (lauric acid), C14:0 (myristic acid), C16:0 (palmitic acid), C18:0 (stearic acid), C20:0 (arachidic acid), C12:1 (lauroleic acid), C14:1 (myristoleic acid), C16:1 (palmitoleic acid), C18:1 (oleic acid), C18:2 (linoleic acid) and C20:4 (arachidonic acid). The resultant type I difference spectra obtained for each fatty acid are shown. Each experiment was performed in triplicate although only one replicate is shown.

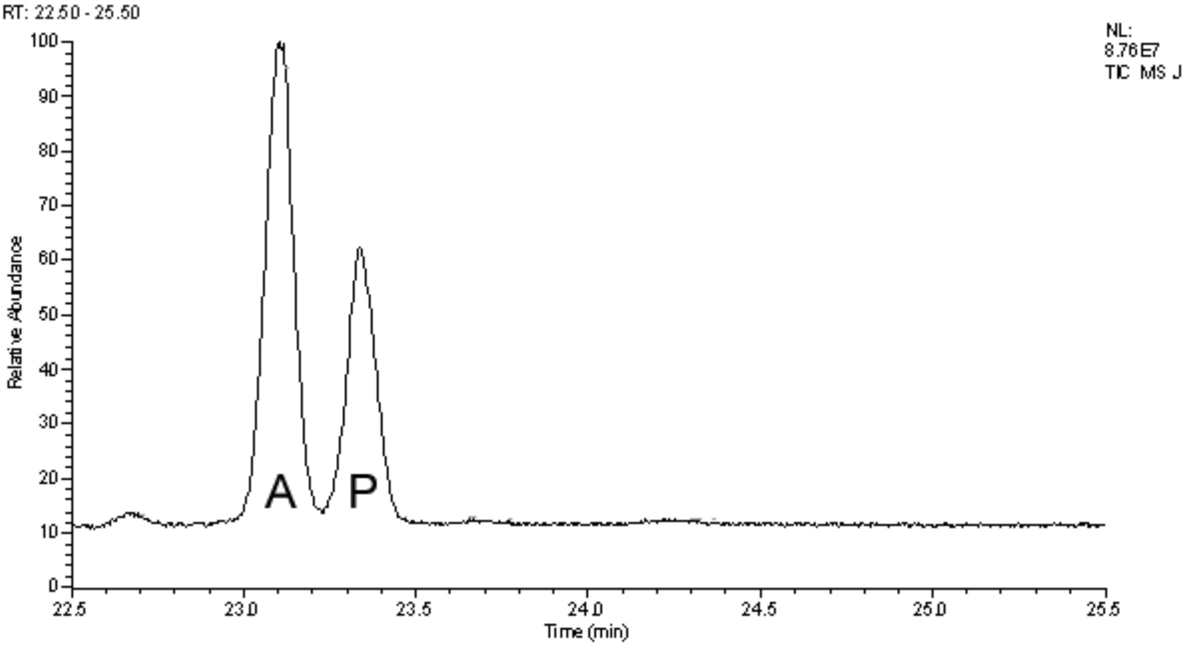
RT: 22.50 - 25.50
NL:
8.76E7
TIC MS J
Relative Abundance
A
P
Time (min)

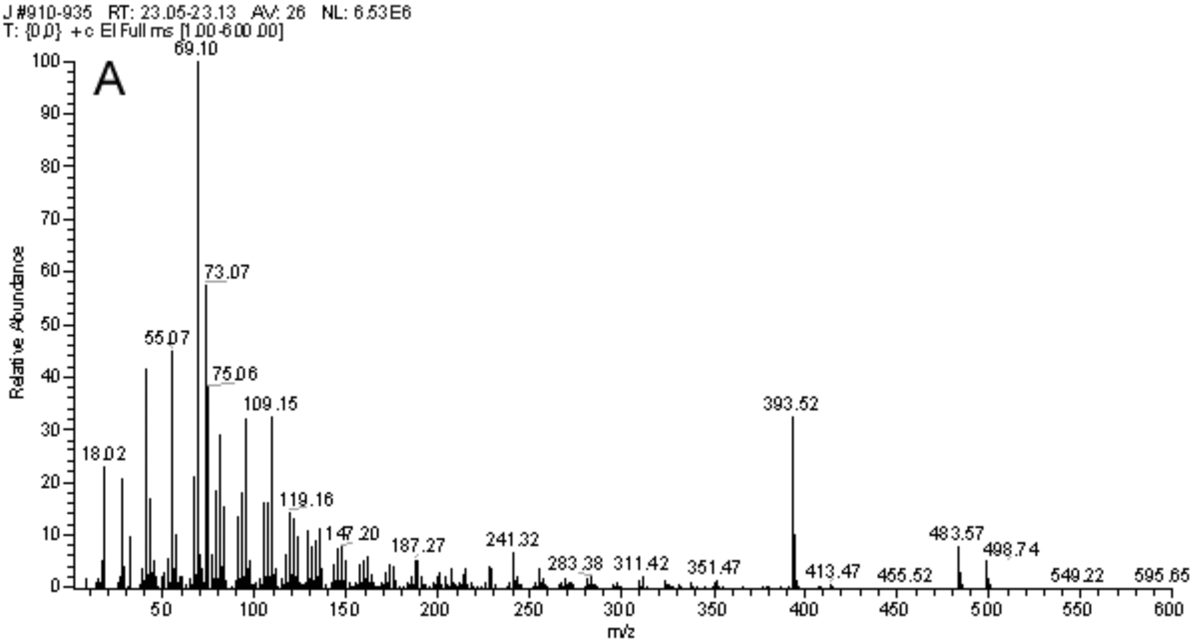
J #910-935 RT: 23.05-23.13 AV: 26 NL: 6.53E6
T: {0,0} + c EI Full ms [1.00-600.00]
A
69.10
73.07
55.07
75.06
109.15
393.52
18.02
119.16
147.20
187.27
241.32
283.38
311.42
351.47
413.47
455.52
483.57
498.74
549.22
595.65
Relative Abundance
m/z

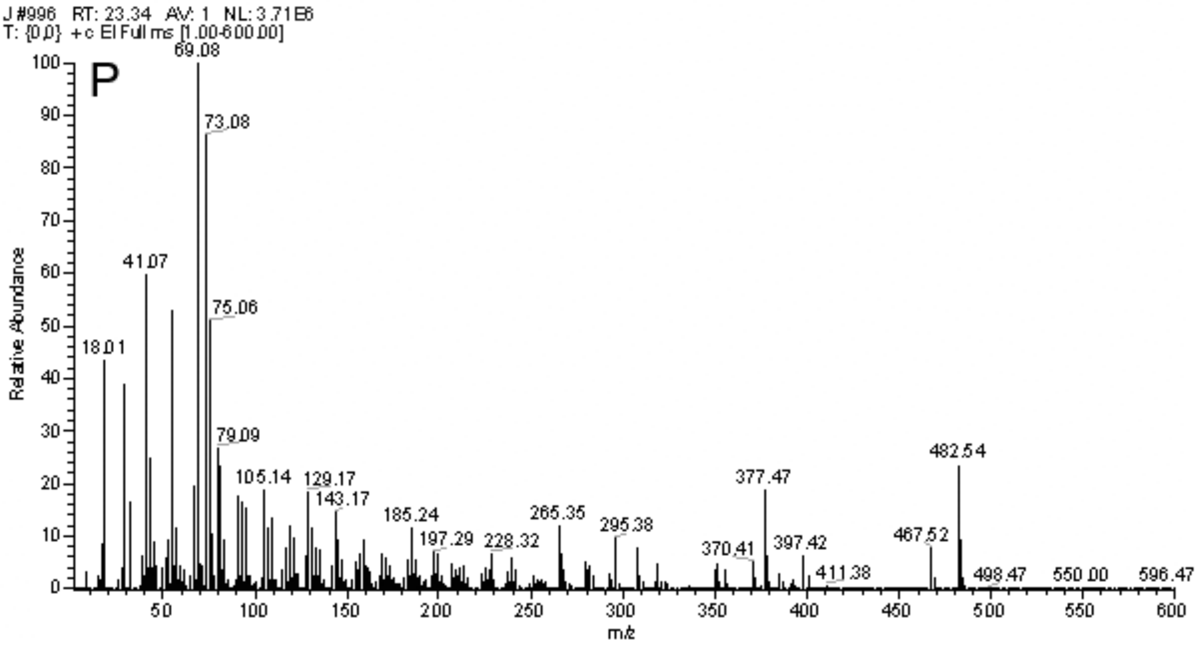
J #996 RT: 23.34 AV: 1 NL: 3.71E6
T: {0,0} + c EI Full ms [1.00-600.00]
P
69.08
73.08
41.07
75.06
18.01
79.09
105.14
129.17
143.17
185.24
197.29
228.32
265.35
295.38
377.47
370.41
397.42
411.38
467.52
482.54
498.47
550.00
596.47
Relative Abundance
m/z

Figure S2. GC/MS analysis of lanosterol demethylation by CYP51. The GC trace for CYP51 metabolites using lanosterol as substrate and the mass fragmentation patterns for the TMS-derivatized lanosterol (A - *m/z* 498) and C14-demethylated lanosterol (P - *m/z* 482) are shown.

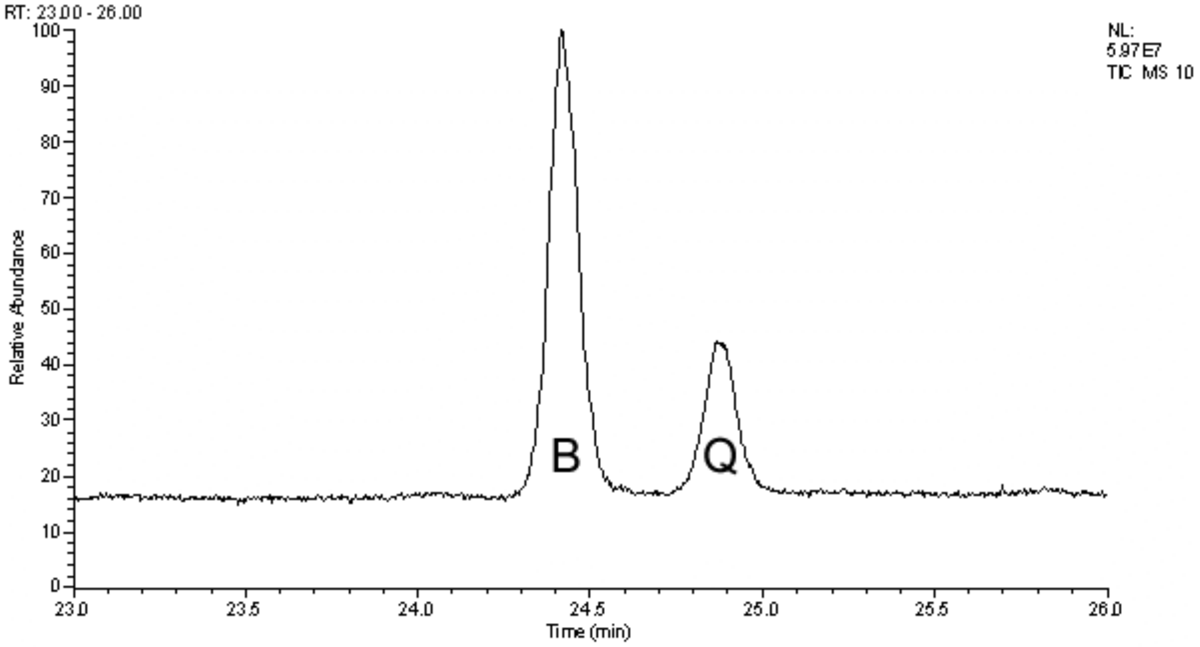
RT: 23.00 - 26.00
NL:
5.97E7
TIC MS 10
Relative Abundance
Time (min)
B
Q

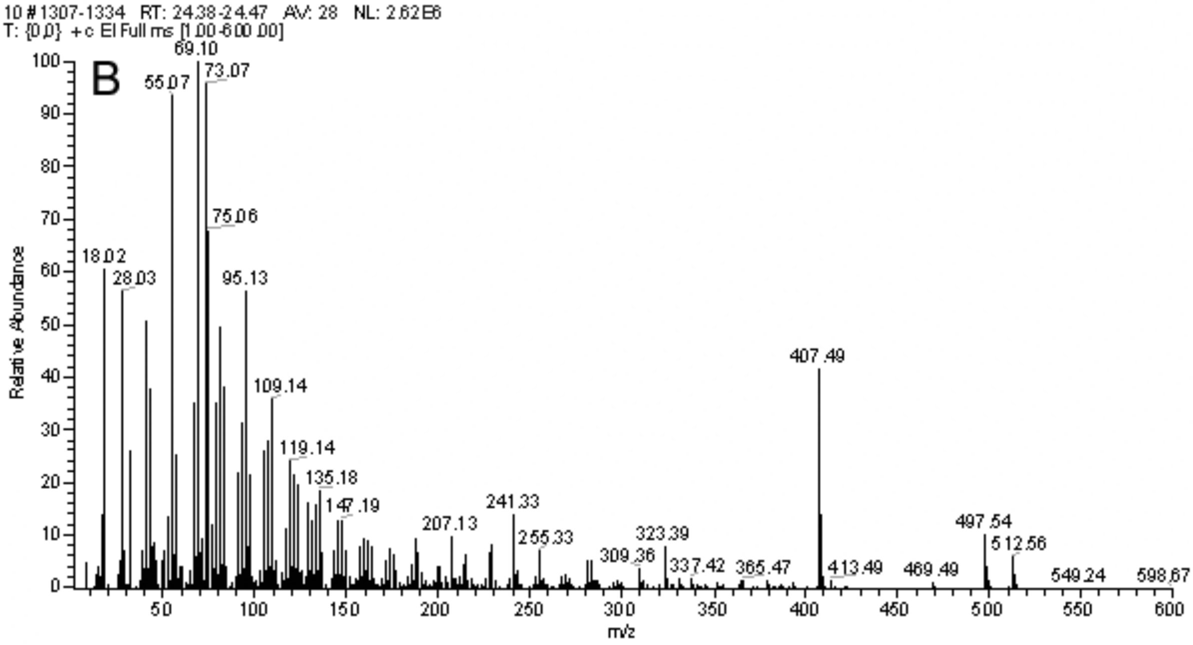
10 #1307-1334 RT: 24.38-24.47 AV: 28 NL: 2.62E6
T: {0,0} +c EI Full ms [1.00-600.00]
B
69.10
55.07
73.07
75.06
18.02
28.03
95.13
109.14
119.14
135.18
147.19
207.13
241.33
255.33
309.36
323.39
337.42
365.47
407.49
413.49
469.49
497.54
512.56
549.24
598.67
Relative Abundance
m/z

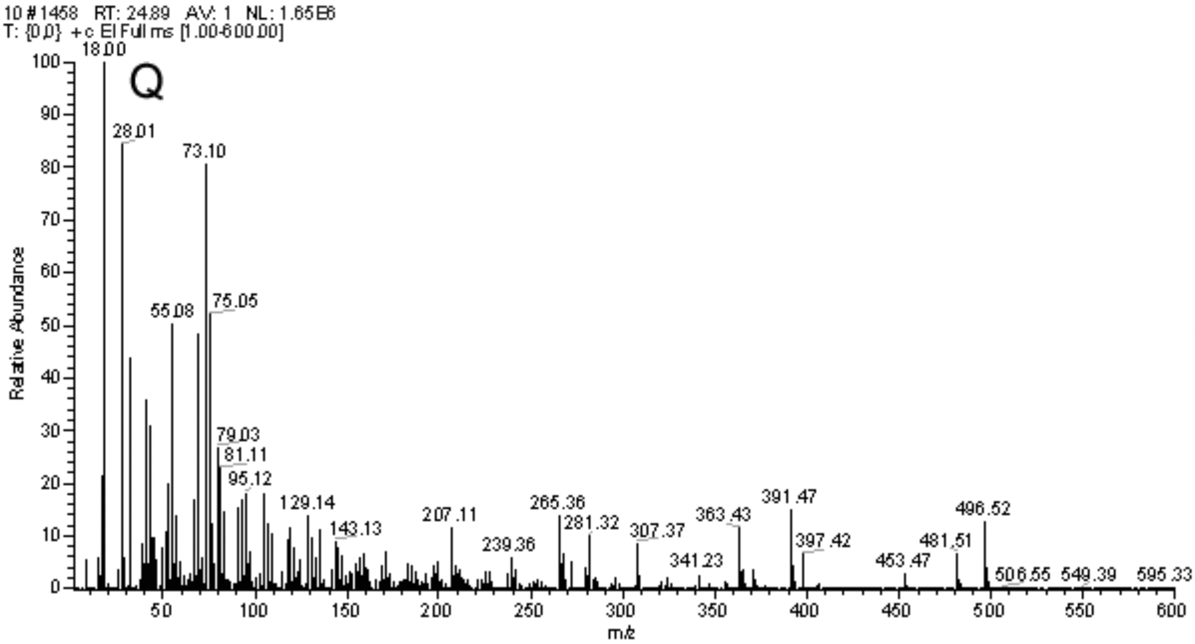
10 #1458 RT: 24.89 AV: 1 NL: 1.65E6
T: {0,0} +c EI Full ms [1.00-600.00]
18.00
Q
28.01
73.10
55.08
75.05
79.03
81.11
95.12
129.14
143.13
207.11
239.36
265.36
281.32
307.37
341.23
363.43
391.47
397.42
453.47
481.51
496.52
506.55
549.39
595.33
Relative Abundance
m/z

Figure S3. GC/MS analysis of eburicol demethylation by CYP51. The GC trace for CYP51 metabolites using eburicol as substrate and the mass fragmentation patterns for the TMS-derivatized eburicol (B - *m/z* 512) and C14-demethylated eburicol (Q - *m/z* 496) are shown.

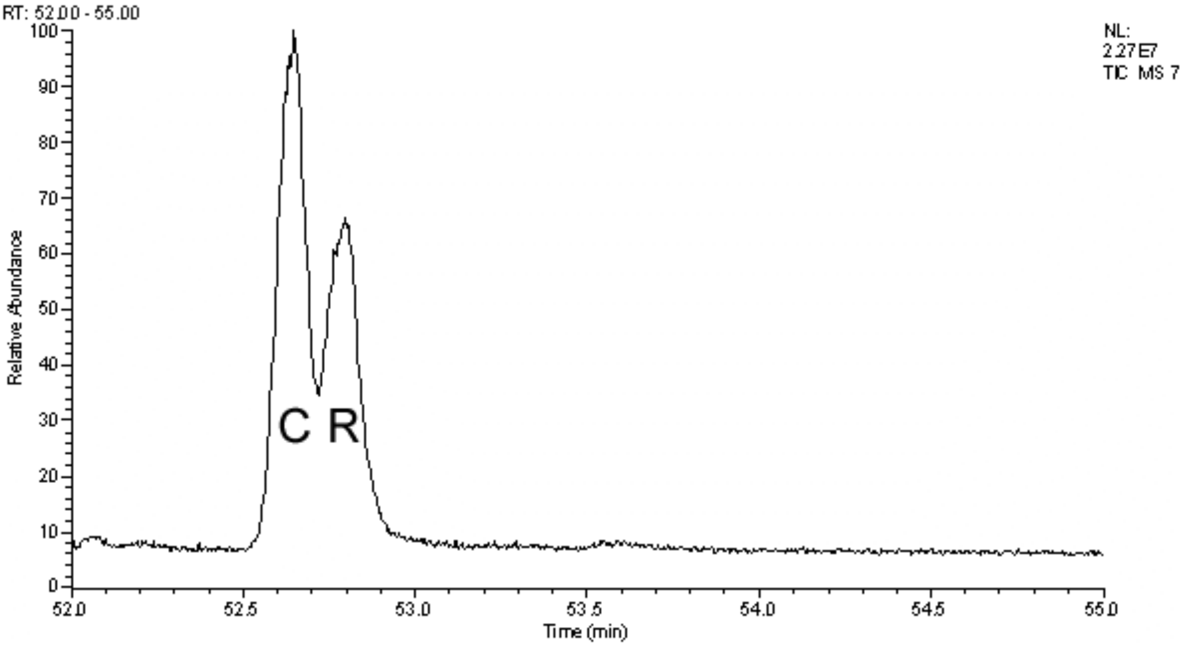
RT: 52.00 - 55.00
NL:
2.27E7
TIC MS 7
Relative Abundance
Time (min)
C
R
0
10
20
30
40
50
60
70
80
90
100
52.0
52.5
53.0
53.5
54.0
54.5
55.0

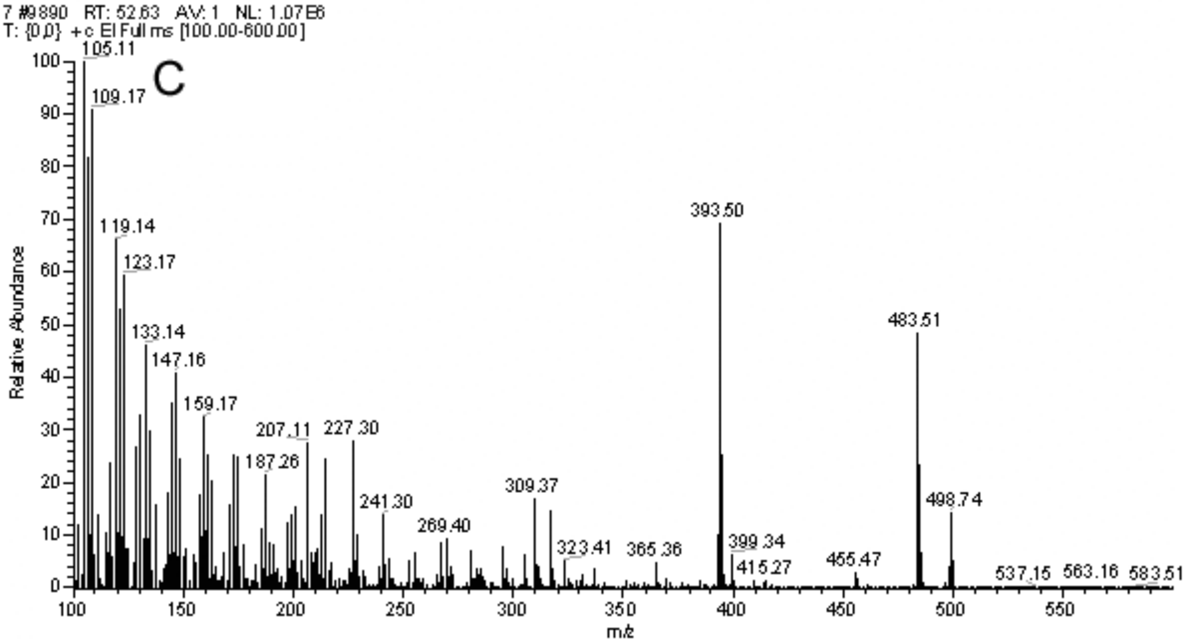
7 #9890 RT: 52.63 AV: 1 NL: 1.07E6
T: {0,0} +c EI Full ms [100.00-600.00]
C
Relative Abundance
m/z
105.11
109.17
119.14
123.17
133.14
147.16
159.17
187.26
207.11
227.30
241.30
269.40
309.37
323.41
365.36
393.50
399.34
415.27
455.47
483.51
498.74
537.15
563.16
583.51
0
10
20
30
40
50
60
70
80
90
100
100
150
200
250
300
350
400
450
500
550

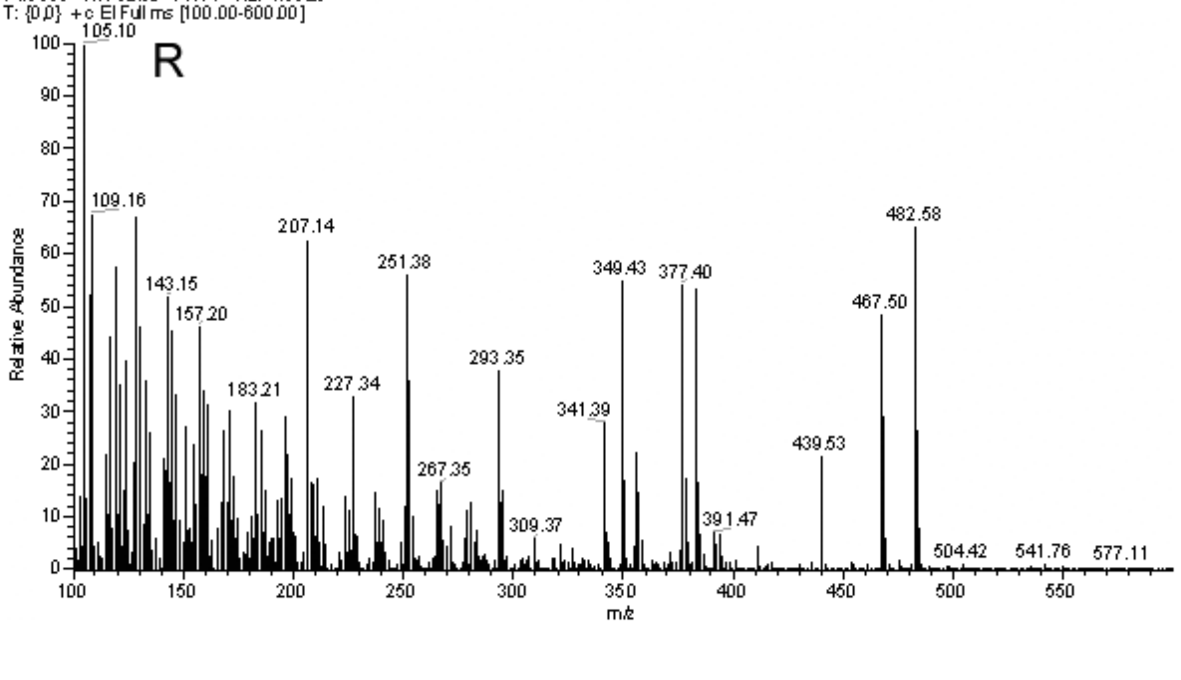
7 #9938 RT: 52.80 AV: 1 NL: 4.95E5
T: {0,0} +c EI Full ms [100.00-600.00]
R
Relative Abundance
m/z
105.10
109.16
143.15
157.20
183.21
207.14
227.34
251.38
267.35
293.35
309.37
341.39
349.43
377.40
391.47
439.53
467.50
482.58
504.42
541.76
577.11
0
10
20
30
40
50
60
70
80
90
100
100
150
200
250
300
350
400
450
500
550

Figure S4. GC/MS analysis of obtusifoliol demethylation by CYP51. The GC trace for CYP51 metabolites using obtusifoliol as substrate and the mass fragmentation patterns for the TMS-derivatized obtusifoliol (C - *m/z* 498) and C14-demethylated obtusifoliol (R - *m/z* 482) are shown. The temperature ramp gradient for the GC of obtusifoliol samples were five-times slower (5°C min^{-1}) than for lanosterol and eburicol to aid separation of products.

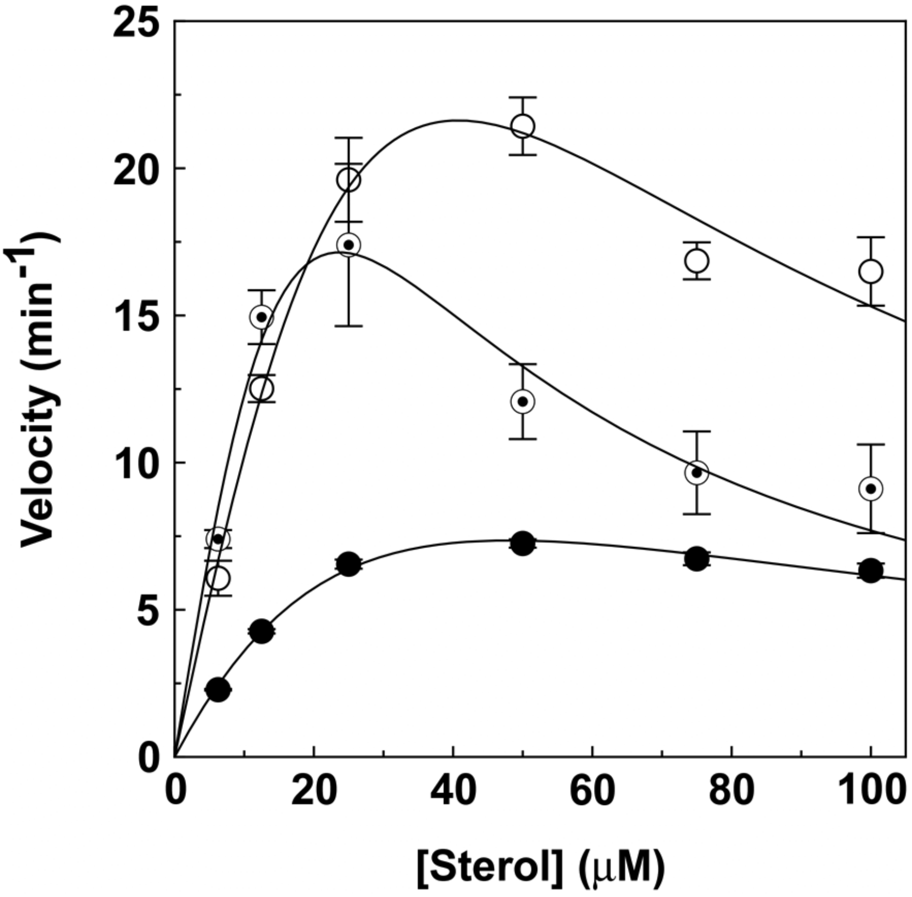

Figure S5. Sterol velocity curves for 0.13 µM *M. globosa* CYP51. Enzyme velocity curves were determined for lanosterol (filled circles), eburicol (hollow circles) and obtusifoliol (bullets) using 0.13 µM CYP51 and 0.5 µM AfCPR1 with 5 min incubation at 37°C. The single substrate inhibition equation [$v = (V_{max}.[S])/\{K_m + [S] + ([S]^2/K_i)\}$] was used to fit the data.

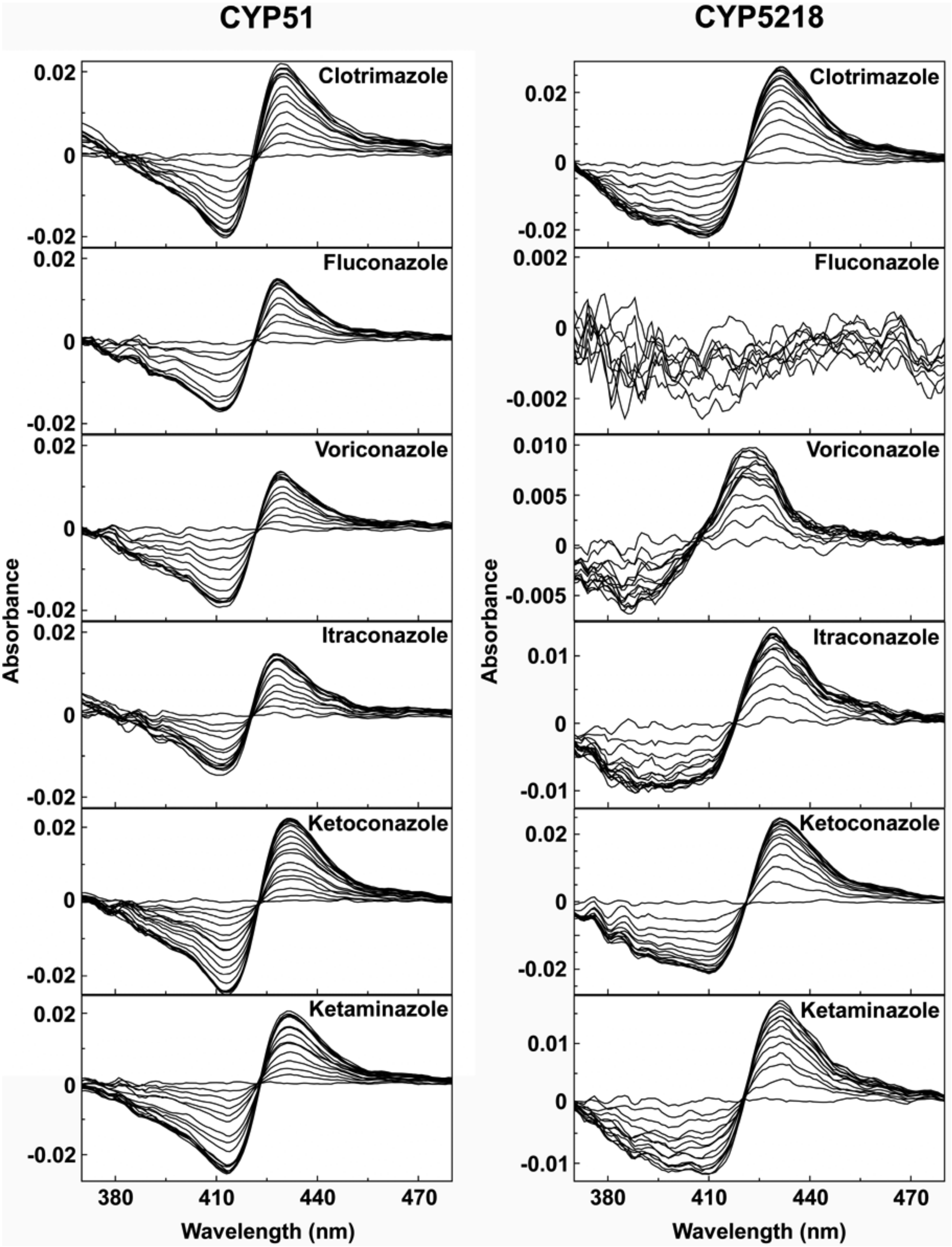

Figure S6. Type II azole binding spectra for *M. globosa* CYP51 and CYP5218. Clotrimazole, fluconazole, voriconazole, itraconazole, ketoconazole and ketaminazole were progressively titrated against 2 μM CYP with the difference spectra determined after each addition of azole. The resultant type II difference spectra obtained for each azole antifungal are shown. Each experiment was performed in triplicate although only one replicate is shown.

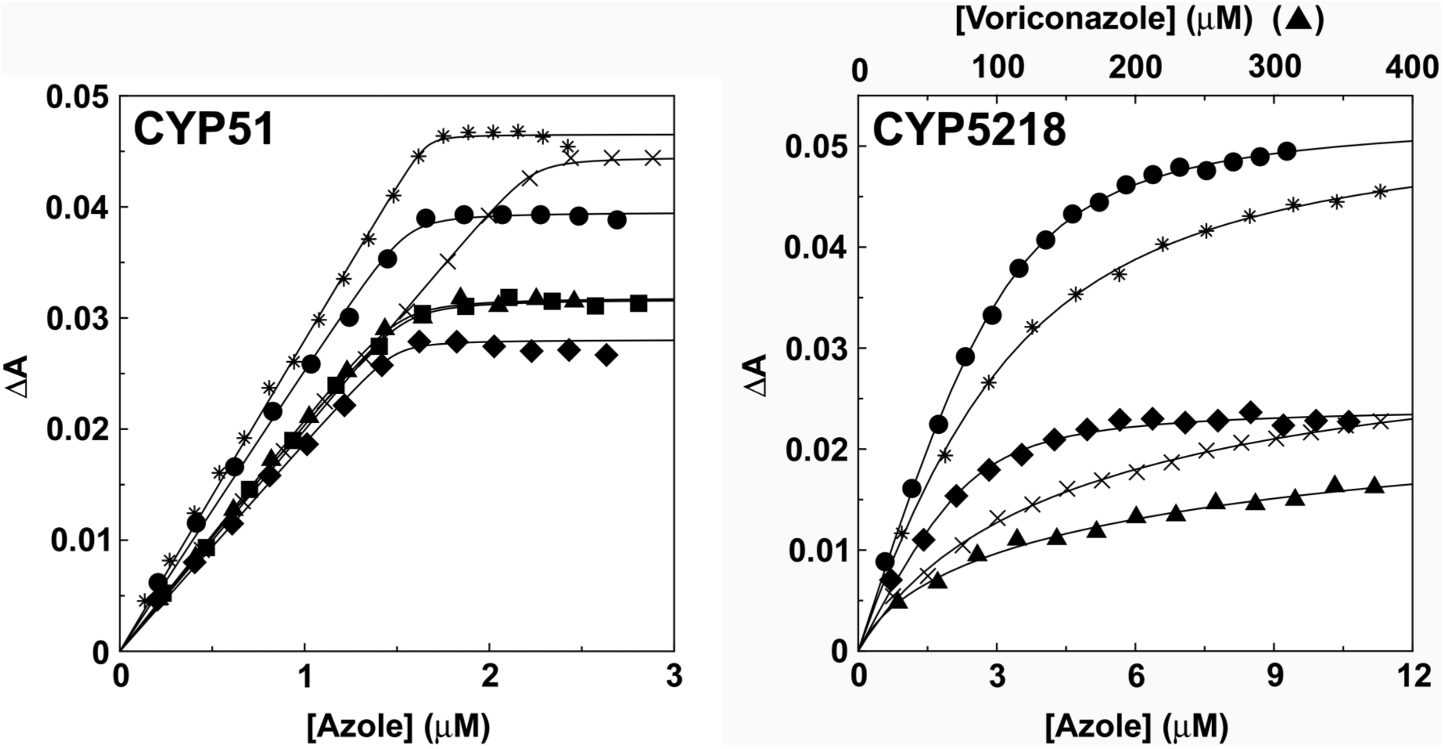

Figure S7. Azole binding saturation curves for *M. globosa* CYP51 and CYP5218. Saturation curves were constructed from the absorbance difference $\Delta A_{peak\text{-}trough}$ of the type II difference spectra (Fig. S2) for clotrimazole (circles), fluconazole (squares), voriconazole (triangles), itraconazole (diamonds), ketoconazole (asterisks) and ketaminazole (crosses). A rearrangement of the Morrison equation[47] was used to fit tight ligand binding and the Michaelis-Menten equation was used where ligand binding was no longer tight. Fluconazole gave no reproducible binding spectra with CYP5218. Each experiment was performed in triplicate although only one replicate is shown.
